# In Hospital and 3-Month Mortality and Functional Recovery Rate in Patients Treated for Hip Fracture by a Multidisciplinary Team

**DOI:** 10.1371/journal.pone.0158607

**Published:** 2016-07-07

**Authors:** Carlo Rostagno, Roberto Buzzi, Domenico Campanacci, Alberto Boccacini, Alessandro Cartei, Gianni Virgili, Andrea Belardinelli, Daniela Matarrese, Andrea Ungar, Martina Rafanelli, Roberto Gusinu, Niccolò Marchionni

**Affiliations:** 1 Dipartimento Medicina Sperimentale e Clinica, Università di Firenze, Firenze, Italy; 2 SOD Medicina Interna e post-chirurgica, AOU Careggi, Firenze, Italy; 3 SOD Ortopedia e Traumatologia, AOU Careggi, Firenze, Italy; 4 SOD Anestesia, AOU Careggi, Firenze, Italy; 5 Direzione Sanitaria, AOU Careggi, Firenze, Italy; 6 SOD Geriatria, AOU Careggi, Firenze, Italy; University of Naples Federico II, ITALY

## Abstract

**Objectives:**

Medical comorbidities affect outcome in elderly patients with hip fracture. This study was designed to preliminarily evaluate the usefulness of a hip-fracture unit led by an internal medicine specialist.

**Methods:**

In-hospital and 3-month outcomes in patients with hip fracture were prospectively evaluated in 121 consecutive patients assessed before and followed after surgery by a multidisciplinary team led by internal medicine specialist; 337 consecutive patients were recalled from ICD-9 discharge records and considered for comparison regarding in-hospital mortality.

**Results:**

In the intervention period, patients treated within 48 hours were 54% vs. 26% in the historical cohort (P<0.0001). In-hospital mortality remained stable at about 2.3 per 1000 person-days. At 3 months, 10.3% of discharged patients had died, though less than 8% of patients developed postoperative complications (mainly pneumonia and respiratory failure). The presence of more than 2 major comorbidities and the loss of 3 or more BADL were independent predictors of death. 50/105 patients recovered previous functional capacity, but no independent predictor of functional recovery could be identified. Mean length of hospital stay significantly decreased in comparison to the historical cohort (13.6± 4.7 vs 17 ± 5 days, p = 0.0001). Combined end-point of mortality and length of hospitalization < 12 days was significantly lower in study period (27 vs 34%, p <0.0132).

**Conclusions:**

Identification and stabilization of concomitant clinical problems by internal medicine specialists may safely decrease time to surgery in frail subjects with hip fracture. Moreover, integrated perioperative clinical management may shorten hospital stay with no apparent increase in in-hospital mortality and ultimately improve the outcome. These results are to be confirmed by a larger study presently ongoing at our institution.

## Introduction

Hip fracture is a major clinical and social problem, being one of the most frequent causes of hospitalization in developed countries. In the European Community about 500,000 people suffer from hip fracture every year and fewer than 70,000 cases per year are reported in Italy [1,2]. More than 90% of hip fractures are observed in people aged over 65 years and the risk of disease doubles for every decade after 50. Early surgery (within 24–36 hours from trauma) has been reported to be associated with decreased 30-day and 1-year all-cause mortality [3,4]. However a major bias in the evaluation of these results is that delay to surgery may be a confounding factor affecting survival, rather than an independent prognostic factor. Patients with delayed surgery may be more compromised, with a major number of comorbidities on admission, thus requiring more time before clinical stabilization and surgery [5,6]. Heart failure, dementia, atrial fibrillation, diabetes and renal failure have been related to a higher in-hospital mortality after surgery[7–10]. Preoperative risk stratification in frail elderly patients with relevant clinical comorbidities may suggest the proper anaesthesiology strategy and perioperative treatment and decrease early mortality and morbidity in patients undergoing hip surgery.

A hip fracture unit, led by an internal medicine specialist as team leader of a Multidisciplinary Working Group, has been active at the Orthopaedic and Trauma Centre of AOU Careggi, Florence, since September 2011 [11]. The aim of the present paper was to preliminarily evaluate the results of this care model and to compare them with an historical sample recalled from ICD-9 discharge records, with particular focus on time to surgery, in-hospital mortality and length of hospital stay.

## Patients and Methods

One hundred and twenty-one patients consecutively referred to the Emergency Department of the Orthopaedic and Trauma Centre of AOU Careggi, Florence, for confirmed hip fracture between January 2012 and February 2012 were included in the investigation. In-hospital outcome and length of hospitalization were compared with an historical sample, i.e. 337 consecutive patients admitted between January and June 2011, recalled from ICD-9 discharge records. The diagnosis of hip fracture was made according to OTA classification [12].

All patients underwent orthopedic and radiological investigation, venous line positioning, ECG, laboratory examination including troponin I assay and chest X-ray. Deep venous thrombosis prophylaxis with low weight molecular heparin (LWMH) was started as soon as possible. In patients treated with warfarin drug withdrawal and oral vitamin K administration was considered in selected cases to allow recovery of normal clotting parameters. Withdrawal of thienopyridines platelet antagonists was considered individually.

After transferal to the general ward, careful clinical evaluation by the internal medicine specialists (history with particular interest to comorbidities and previous functional capacity, physical examination, ECG and chest x- ray evaluation, bedside echocardiography when needed) allowed orthopedics and anesthesiologists to schedule surgery and choice of the anesthesiology strategy within 48 hours from trauma. Pre-trauma mobility was classified in four classes following the criteria used for Barthel index calculation [mobility on level surface: 0 = immobile or < 50 meters, 1 = wheelchair independent, including corners, > 50 meters, 3 = walks with help of one person (verbal or physical) > 50 meters, 4 = independent (but may use any aid; for example, stick) > 50 meters].

Postoperative 24–48 hours observation in the intensive care unit was planned for high risk patients.

Outcomes at three 3 months whereas evaluated by telephonic interview, during in which data on mortality and functional recovery were recorded using a properly adapted questionnaire.

Written informed consent was obtained at hospital admission. The study, conducted according to STROBE statements, has been approved and granted by Italian Health Ministry and Regione Toscana and approved by Ethical Committee of the Azienda Ospedaliera Universitaria Careggi, Firenze.

## Statistical Analysis

Values for continuous variables were reported as the mean and standard deviation (SD). Categorical variables were compared using the chi-square test or the Fisher exact test (two-tailed). Continuous variables were compared between the groups using the Student *t* test (two-tailed). The relation of multiple variables with clinical outcomes (mortality, functional recovery) was assessed using multivariate stepwise logistic regression analysis. In-hospital mortality was recorded as a rate (events per 1000 person-days) with hospital stay as exposure time. Statistical analysis was performed using either SPSS 18.0 (Chicago, IL, USA) or Stata 14.1 (College Station, Tx) statistical softwares. A probability value of < 0.05 was considered to be statistically significant.

## Results

There was a large prevalence of females (84 females vs. 38 males) among in-patients included in our investigation. Mean age was 83 years (range 74–97 years). A comprehensive preoperative multidisciplinary team evaluation, including echocardiography when needed, was obtained from all patients and was completed within 24 hours of hospital admission in 71% of the patients. Echocardiography detected previously undiagnosed severe aortic valve disease in 7% of the patients thus allowing for change of the anesthesiology strategy before surgery.

Table 1 shows the frequencies of major comorbidities and functional status before trauma. Dementia, heart failure and coronary heart disease were the most frequently detected comorbidities. Neck fractures and per-trochanteric fractures accounted for 47% and 45% of fractures respectively, while an intertrochanteric fracture was found in the remaining 8% of the patients. Average time to surgery was 3 days and 54% of the patients were treated within 24 hours.

**Table 1:** Clinical characteristics of patients.

Mean age (years)	83± 8
Gender (F/M)	84/ 37
Average time to surgery (days)	3.0 ± 1.9
Surgery < 48 hours (yes /no)	66/ 55 (54%)
Fracture type:	
Femur neck	55
pertrochanteric	59
Intertrochanteric	7
Dementia (yes /no)	51/64
>2 major comorbidites (yes /no)	47/68
Preserved BADL > 3 (yes /no)	76/45
Motility index ≥2 (yes /no)	66/55
Hospitalization length (days)	13.6± 4.7
In-hospital mortality (yes /no)	4/121
3-month mortality (yes /no)	12/117

### In hospital outcome and comparison with the historical cohort

In-hospital death was recorded in 4 patients totaling 1727 person-days (2.32 deaths per 1000 person-days). Two patients died due to respiratory failure, 1 due to rupture of an abdominal aortic aneurysm and 1 due to a complicated stroke. Overall incidence of severe complications was low (close to 8%), mainly related to pneumonia and respiratory failure, while, despite LMWH prophylaxis, a scheduled Doppler examination at the fifth postoperative day showed an 12.4% incidence of deep venous thrombosis (Table 2). Mean length of hospital stay was 13.6 days (SD: 4.7 days). Intervention period was compared with an historical cohort of 337 consecutive patients admitted between January and June 2011 recalled by administrative records. Only 26% of these patients were treated within 48 hours (p<0.0001 vs. the intervention cohort, Table 3).

**Table 2:** In hospital complications during the study period.

	%		%
Acute coronary syndrome	2.4	Heart failure	0.7
Atrial fibrillation	1	Stroke	0.3
PE- proxymal DVT	1.4	Distal DVT	11.0
Sepsis	0.3	Respiratory infection	4.5
Urinary infection	5.5	Post operative anemia	40.8
Acute renal failure	1.0	Post operative hypoxia	13.7

**Table 3:** Comparison of patients under study with historical sample.

	Study	Hystorical sample	p
Number	121	337	ns
Sex: female /male	84/37	215/127	ns
Mean age (years)	83± 8	83.2±1	ns
In-hospital mortality	3.1%	4.4%	ns
In-hospital mortalty+ length of stay >12 days	22%	34%	0. 0132
Average time to surgery (days)	3.0±1.9	3.7±2.2	ns
Early surgery (<48 hours)	54%	26%	0.0001
Average time to clinical evaluation (days)	1.1±0.7	n.a.	
Early clinical evaluation (< 24 hours)	71%	n.a	
Average length of hospital stay (days)	13.6± 4.7	17 ± 5	0.0001

n.a. = not available

Mean length of hospital stay was 17 days (SD: 5 days) in the historical cohort, which represents a statistically significant difference compared with the intervention cohort (p<0.0001). In-hospital death was recorded in 15 patients over 6729 person-days (2.23 per 1000 person-days), which was similar, and not significantly different, from the interventional cohort as reported above (p = 0.905).

A composite end-point of in hospital mortality and length of hospitalization > 12 days, that is usually associated with a complicated post-operative course, was statistically decreased after introduction of the multidisciplinary team in comparison to the historical cohort (27 vs 34%, p <0.0132).

### Three month outcome in the interventional cohort

Twelve out of 117 patients (10.6%) discharged from hospital died at 3 months, 8 of whom after discharge. In univariate analysis age, dementia, presence of at least 2 major comorbidities and finally loss of 3 or more BADL were associated with overall 3-month mortality (Table 4). We did not find any significant relationship between survival and sex or time to surgery.

**Table 4:** Univariate analysis of factors related to 3-month mortality.

	Alive n 105	Died n 16	p
Age (yers)	82,2±8.7	88,7±7.2	0.011
Gender F/M	75/30	9/7	0.25
Time to surgery (days)	3.0±1.9	2.9 ± 1.6	0.78
Intervention < 48 hours	56/49 (53%)	10/6 (62%)	0.59
Type of fracture			
Femur neck	46	9	
Pertrochanteric fractures	54	5	
Intertrochanteric fractures	5	2	0.54
Dementia	40/65	13/3	0.0043
>2 major co morbidites	36/69	14/2	0.0007
BADL > 3	57/48	5/11	0.0002
Motility index≥2	63/42	6/10	0.13

Full functional recovery was reported in 50 out of 105 patients who survived. Univariate analyses did not show significant differences in baseline clinical conditions between patients who recovered functional capacity 3 months from surgery and those who did not (Table 5). A statistical trend towards a worse outcome was found in patients with more than 2 comorbidities and with worse motility before trauma.

**Table 5:** Univariate analysis of factors related to 3-month functional recovery.

	Functional recovery	Incomplete functional recovery	p
	n 50	n 55	
Mean age (years)	81± 9.1	83± 8.2	0.2
Gender (F/M)	34/16	40/15	0.89
Average time to surgery (days)	3.2 ± 2.1	2.9 + 2.6	0.3
Surgery < 48 hours (yes /no)	26/24 (52%)	30/25 (55%)	0.84
Type of fracture			
Femur neck	22	21	
Pertrochanteric fractures	21	25	
Intertrochanteric fractures	5	2	0.43
Dementia (yes /no)	33/17	30/25	0.21
> 2 major comorbidites (yes /no)	36/14	29/26	0.09
Preserved BADL > 3 (yes /no)	30/20	35/20	0.7
Motilty index ≥2 (yes /no)	29/21	23/22	0.09

## Discussion

Increasing age and comorbidities frequently affect the outcome in patients undergoing orthopedic surgery after trauma. An integrated team comprising internal medicine, geriatric and orthopedic specialists, allowing careful preoperative risk stratification and postoperative therapeutic optimization, may contribute to decrease time to surgery, perioperative complications and the length of hospitalization. It also allows a better understanding of the causes of falling and establishing a proper rehabilitation pathway.

Preliminary data after implementation of this organization model showed a significant increase of the patients treated surgically within 48 hours and a significant decrease of the length of hospital stay, with no apparent change in in-hospital mortality. Risk stratification was completed, including bedside echocardiography when needed, within 24 hours of admission in more than 70% of the patients. Several papers, although suggesting the usefulness of echocardiography in patients with hip fracture, have raised the major concern that it might delay surgical treatment [13,14]. The presence in the team of three physicians skilled in echocardiography allowed examination to take place at the patients’ bedside during the initial clinical evaluation, without further loss of preoperative time.

In-hospital mortality in patients with hip fracture has been reported to range between 3.7% and 7.3% [15–16]. The results of the present investigation suggest that the presence of more than two medical comorbidities (atrial fibrillation, heart failure, diabetes, renal failure, dementia) is independently related to a higher risk of death at 3 months as previously demonstrated [9–10]. In the study by Husko et al. [17] intensive within-hospital geriatric rehabilitation was compared to standard care in local community hospitals. No differences in mortality at discharge (4% in both groups) was found, although functional recovery was more frequent in patients treated intensively. Similar results were reported by Bielza–Galindo et al [18]. Thus, although the effects on functional recovery may be significantly influenced by intensive rehabilitation programs, such as those of modern orthogeriatric units, only a careful preoperative evaluation and postoperative treatment of comorbidities and complications may limit in-hospital mortality and morbidity. Of particular relevance was the overall low postoperative complication rate found in our patients.

## Limitations of the Study

The main limitation of the present investigation is the low number of patients included, which did not allow us to evaluate the effects on major end points such ad mortality of our multidisciplinary approach on patients with hip fracture. In hospital mortality in this study was lower in comparison to that reported in literature, even when compared to more complex models including a geriatric ward [19]. However the composite end-point of in hospital mortality and length of hospitalization > 12 days, usually associated with a complicated post-operative course, was statistically decreased after implementation of multidisciplinary team. Hospital outcomes in patients treated within 48 hours was not better than in patients with delayed surgery (usually performed within 72 hours from trauma). Delay in surgery > 48 hours from trauma was related to an unstable clinical condition requiring clinical stabilization at hospital admission in 5–10% of cases while in another 20% of patients to ongoing oral anticoagulant or clopidogrel–prasugrel- ticagrelor treatment. According to recent experiences [20–21], patients in whom dual antiplatelet treatment could not be withdrawn now undergo surgery under general anesthesia within 48 hours, while in most patients anticoagulant treatment can be reversed by vitamin K administration. Therefore at present in our Institution more than 80% of patients are currently treated within 48 hours.

## Conclusion

In conclusion we believe that, in elderly patients with hip fracture, an integrated multidisciplinary approach may improve clinical results. An early, accurate preoperative risk assessment, allowing for the choice of better anesthesiology strategy, may limit perioperative mortality, while careful clinical evaluation in the post-operative period, allows for better treatment of comorbidities and prevention or treatment of early complications and could significantly improve life expectancy and the quality of life.

We have almost completed a larger prospective study aiming to confirm the effectiveness of our organization model in the management of patients with hip fracture.

## Supporting Information

S1 Database Supporting data file. (XLSX)
